# Impact of Climatic Factors on the Temporal Trend of Malaria in India from 1961 to 2021

**DOI:** 10.3390/tropicalmed9120309

**Published:** 2024-12-19

**Authors:** Muniaraj Mayilsamy, Rajamannar Veeramanoharan, Kamala Jain, Vijayakumar Balakrishnan, Paramasivan Rajaiah

**Affiliations:** 1 ICMR-Vector Control Research Centre Field Station, No. 4, Sarojini Street, Chinna Chokkikulam, Madurai 625 002, Tamil Nadu, India; 2 Department of Biotechnology, Madha Enginerring College, Chennai 600 069, Tamil Nadu, India; 3 ICMR-Vector Control Research Centre, Indira Nagar, Puducherry 605 006, India

**Keywords:** India, climatic factors, temporal trend, malaria

## Abstract

Malaria remains a significant public health problem in India. Although temperature influences Anopheline mosquito feeding intervals, population density, and longevity, the reproductive potential of the Plasmodium parasite and rainfall influence the availability of larval habitats, and evidence to correlate the impact of climatic factors on the incidence of malaria is sparse. Understanding the influence of climatic factors on malaria transmission will help us predict the future spread and intensification of the disease. The present study aimed to determine the impact of temporal trend of climatic factors such as annual average maximum, minimum, mean temperature, and rainfall on the annual incidence of malaria cases in India for a period of 61 years from 1961 to 2021 and relative humidity for a period of 41 years from 1981 to 2021. Two different analyses were performed. In the first analysis, the annual incidence of malaria and meteorological parameters such as annual maximum, minimum, and mean temperature, annual rainfall, and relative humidity were plotted separately in the graph to see if the temporal trend of climatic factors had any coherence or influence over the annual incidence of malaria cases. In the second analysis, a scatter plot was used to determine the relationship of the incidence of malaria in response to associated climatic factors. The incidence of malaria per million population was also calculated. In the first analysis, the annual malaria cases showed a negative correlation of varying degrees with relative humidity, minimum, maximum, and mean temperature, except rainfall, which showed a positive correlation. In the second analysis, the scatter plot showed that the rainfall had a positive correlation with malaria cases, and the rest of the climatic factors, such as temperature and humidity, had negative correlations of varying degrees. Out of the total 61 years studied, in 29 years, malaria cases increased more than 1000 square root counts when the minimum temperature was at 18–19 °C; counts also increased over a period of 33 years when the maximum temperature was 30–31 °C, over 37 years when the mean temperature was 24–25 °C, over 20 years when the rainfall was in the range of 100–120, and over a period of 29 years when the relative humidity was at 55–65%. While the rainfall showed a strong positive correlation with the annual incidence of malaria cases, the temperature and relative humidity showed negative correlations of various degrees. The increasing temperature may push the boundaries of malaria towards higher altitude and northern sub-tropical areas from the southern peninsular region. Although scanty rainfall reduces the transmission, increases in the same would increase the malaria incidence in India.

## 1. Introduction

Globally, there were an estimated 249 million cases of malaria in 85 malaria-endemic countries in 2022, with an estimated 608,000 malaria deaths [1], and the death toll has not improved since 2015 [2]. Nearly half of the world’s population is threatened by malaria [3]. Parasites and mosquitoes that are resistant to current interventions are spreading fast across the globe [2]. India alone accounts for 66% of total malaria cases in the WHO Southeast Asia Region, which accounts for 2% of global malaria cases [1]. In contrast to the increasing global malaria cases and deaths from 214 million and 438,000 in 2015, to 227 million and 558,000 in 2019, and 241 million and 627,000 in 2020 [4], respectively, the number of malaria cases and deaths in India steadily declined from 2.03 million and 931 in 2000 to approximately 161,000 and 90 in 2021, respectively [5,6]. Despite continuous decline, malaria remains a major public health problem in India, with almost 95% of the country’s population living in malaria-endemic areas and more than 80% of the malaria cases reported from 20% of the population living in tribal hills [7]. Apart from major challenges such as insufficient surveillance, slow and aggregated data reporting, complex epidemiological features that include involvement of different species of parasites, vectors, susceptible human living in rural and forested areas [8,9], the development of resistance in parasites to antimalarial drugs, the development of resistance in vectors to the insecticides [10], and a lack of available health care systems with diagnostic and treatment facilities [11], climate change due to global warming has not gained the attention it deserves in terms of its impact on malaria control. Although evidence to correlate climatic factors such as temperature, precipitation, and humidity on malaria transmission is available, the long-term impact on the trend of malaria incidence is scanty [1,12]. Temperature affects the feeding intervals, population density, and longevity of Anopheles mosquitoes [13,14,15,16], and the reproductive potential of malaria parasites [16] and rainfall affect the availability of larval habitats [17]. Moreover, changes in climatic conditions and their extremes can generate unexpected changes in the mosquito vector population, with consequent effects on public health [18,19]. Apart from having a direct influence, climate change threatens to derail progress in global health by affecting livelihoods, increasing the risks of harmful exposure to parasites and vectors, overburdening health systems, and widening existing inequalities. Thus, climate change is not just a singular threat but a major multiplier of other threats [1]. Therefore, understanding the impact of climatic factors and climate changes on the occurrence of malaria is of great interest to public health [20,21], which would help to predict the future spread and intensification of the disease [22]. A long-term time-series analysis of climatic factors and malaria cases can demonstrate the influence of climatic factors on the occurrence of malaria cases. In the present study, the temporal trends of annual maximum, minimum, mean temperature, and rainfall from 1961 to 2021 and relative humidity from 1981 to 2021 were compared with the annual incidence of malaria cases in India to determine the impact of climatic factors on the occurrence of malaria cases in India. To determine the current burden of malaria in India, the incidence of malaria per million of the population was also calculated. The present study will provide information on how the changes in annual climatic factors for a period of six decades correlates with the incidence of malaria in India to indirectly elucidate the influence of climatic factors on the incidence of malaria.

## 2. Materials and Methods

The data on the annual incidence of malaria for the period of six decades, from 1961 to 2021, were collected from the health statistics of India, health information of India, and the National Health Profile, published by the Central Bureau of Health Intelligence, Government of India [23]. The meteorological data, such as the annual average maximum, minimum, and mean temperature and rainfall, were collected from the India Meteorological Department, Pune [24], data.gov [25], the Government of India, and Statista [26], and the annual average relative humidity was collected from Statista. All climatic data were compared with malaria cases from 1961 to 2021 except relative humidity, for which the data were compared from 1981. While the annual mean temperature and rainfall data were available from 1961, the data on humidity were only available from 1981. Two different analyses were performed; one is used to compare the temporal trend of climatic factors and annual incidence of malaria cases, whereas the other is used to find out the relationship between the annual climatic factors and the annual incidence of malaria. In the first analysis, the annual incidence of malaria cases and climatic factors such as maximum, minimum, and mean temperature, annual rainfall, and relative humidity were plotted separately in the graph to see if the temporal trend of climatic factors had any visible association with the incidence of malaria cases. A polynomial trendline at order 6 that showed a maximum R^2^ value was drawn for malaria cases and each of the climatic factors. The polynomial trendline is a curved line that can be used to analyze data that fluctuate, such as increases and decreases over a large data set. The higher the R^2^ value (from the order 1–6), the more it mimics the exact trend.

In the second analysis, scatter plots were drawn to determine the impact of climatic factors on the occurrence of malaria cases. In the scatter plot, the square root value of malaria cases was taken on the *y*-axis, and climatic factors were taken individually on the *x*-axis. Each dot in the graph represents the square root value of total malaria cases in a particular year corresponding to the respective climatic factors. A square root count of 1000 is equal to 1 million cases (1000 × 1000 = 1 million). A cutoff line was drawn ad libitum at a square root count of 1000 (=1 million) malaria cases to separate the malaria incidences that fall above or below the line. The annual square root count of malaria cases falling above the cutoff line is marked in red, and cases falling below the cutoff line are marked in blue to show the range of climatic factors that attract more square root counts of cases that fall above or below the cutoff line. For each climatic factor under analysis, a trendline was drawn to note the trend of cases towards increasing climatic factors. The Pearson correlation coefficient was calculated for both square root counts of malaria cases vs. each of the climatic factors. The *p*-value, R^2^-value, and *n*-value were also determined. The data range was also tabulated. The incidence of malaria cases per million population was also calculated from the total number of annual malaria cases with corresponding populations for the years under study to see the actual trend of malaria over the study period from 1961 to 2021. The population data for the study period were collected from the United Nations’ World Population Prospects 2024 [27].

## 3. Results

**Figure 1:**
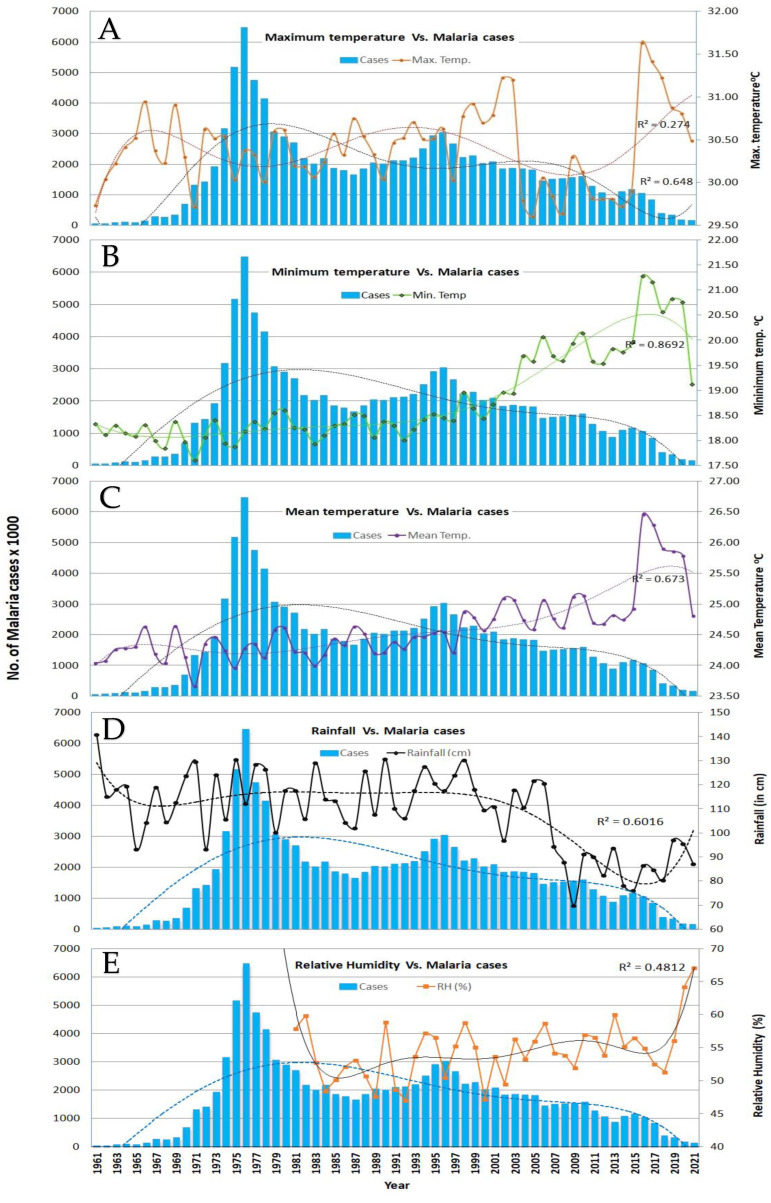
The temporal trend of annual incidence of malaria (blue bars) in India was compared with the temporal trend of annual average maximum temperature (**A**); minimum temperature (**B**); mean temperature (**C**); rainfall (**D**); and relative humidity (**E**). The R^2^ values, shown in dotted lines, show the reliability of the trendline with the actual trend.

In the first analysis, the temporal trend of annual malaria cases in India from 1961 to 2021 (Figure 1) showed that the number of cases in 1961 was the lowest, at 49,151, with an all-time peak of 6,467,215 in 1976. The annual incidence of malaria showed a declining trend until 1987, when the number of cases was 1,663,284, followed by an increase in annual cases again and reaching another peak of 3,035,588 cases in 1996. After that, the number of cases showed a continuous decline until 2021, with 158,326 cases. While the trendline analysis of the minimum temperature with an R^2^ value of 0.8692 (Figure 1B) and mean temperatures with an R^2^ value of 0.673 (Figure 1C) showed an increasing trend in both, the maximum temperature with an R^2^ value of 0.274 (Figure 1A) and relative humidity with an R^2^ value of 0.4812 (Figure 1E) showed a fluctuating trend throughout the study period. In contrast, with an R^2^ value of 0.6016, rainfall (Figure 1D) has shown a decreasing trend since 1961. The temporal trend of malaria with climatic factors and their trendlines from 1961 to 2021 showed that the maximum, minimum, and mean temperatures and humidity showed negative correlations, and rainfall showed a positive correlation. Statistical analysis of the square root of malaria cases and climatic factors (Table 1) showed that, except for rainfall, all other climatic factors showed negative relationships with the annual incidence of malaria.

**Figure 2:**
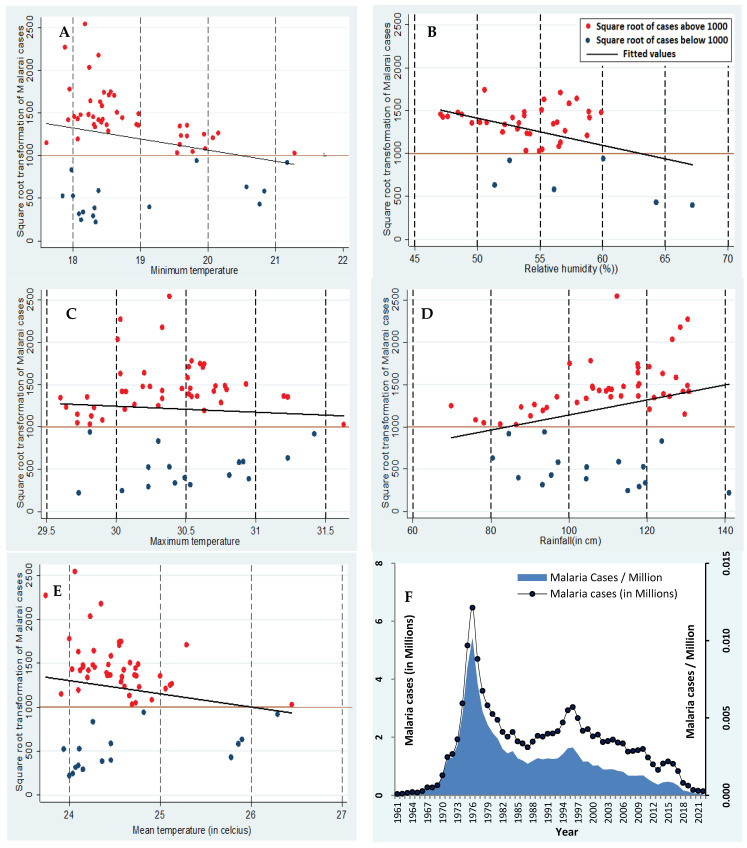
Distribution of the square root of malaria cases in a particular year with the corresponding average annual climatic factor (**A**–**E**). The red dots show the square root count of cases above 1000, and the blue dots show the square root count of cases below 1000. The trendline shows the pattern of the square root count of malaria cases to the corresponding climatic factors. The incidence of malaria cases (in a million population) vs. the total number of reported malaria cases in India is presented in (**F**).

In the second analysis, the scatter plots showed that the annual average rainfall had a strong positive correlation with the annual incidence of malaria, whereas the other four climatic factors, namely minimum, maximum, mean temperature, and humidity, showed negative correlation (Figure 2A–E). In the graphical representation of Figure 2A–E, the dots that fall above and below the cutoff line of 1000 square root counts of malaria cases for each variable of the climatic factor were counted and tabulated (Table 2). Out of the total 61 years analyzed, over a period of 29 years, malaria cases of more than 1000 square root counts were reported when the minimum temperature was 18–19 °C. Similarly, more than 1000 square root counts of malaria cases were reported over a period of 33 years when the maximum temperature was 30–31 °C, over 37 years when the mean temperature was 24–25 °C, and over 20 years when the rainfall was in the range of 100–120 cm. When the relative humidity was 55–65%, more than 1000 square root counts of malaria cases were reported over a period of 29 years. The regression line showed the square root counts of malaria cases except for rainfall, which showed an increasing trend. The rest of the climactic factors, such as minimum, maximum, mean temperature, and relative humidity, showed decreasing trends of varying degrees. The dots that fall below the cutoff line of 1000 square root counts of cases are marked in blue, and those above the cutoff line are marked in red. Overall, the second analysis showed that the most favorable fractions of the climatic factors, which are 18–19 °C minimum temperature, 24–25 °C mean temperature, 30–31 °C maximum temperature, humidity between 50% and 60%, and rainfall between 100 and 140 cm, highly supported the likelihood of an increase in the annual incidence of malaria. Despite the continuously declining annual incidence of malaria from 1996, the annual incidence of malaria in 2022 was far higher than that in 1961 (49,151 in 1961 to 146,559 in 2022). However, the incidence of malaria per million population in 1961 was 0.00011, and the incidence was 0.00010 in 2022 (Figure 2F). This is mainly because of the 2.2-fold increase in population, which is higher than the 1.98% increase in cases during the period from 1961 to 2022.

In India, malaria cases are reported throughout the year, as the right combination of average temperature, rainfall, and humidity persists one or other parts of the country in all seasons throughout the year.

## 4. Discussion

The influence of climate on the transmission of malaria continues to be a subject of debate [28]. While some studies suggested that climate change may intensify the malaria transmission in the current endemic areas or reemergence of the disease in areas which controlled transmission [18,29,30] or eliminated the disease in the past [30,31], others contradict this view, saying that when comparing with other determinants, climate change plays a marginal role in malaria transmission [28,32,33,34,35,36,37,38] and that climate change may even reduce malaria transmission [39]. As the right combination of average temperature, rainfall, and humidity persists across the country over all seasons, malaria cases are reported throughout the country. Therefore, studying the annual variation in climate would be more informative than focusing on seasonal climatic variations on the occurrence of malaria. Although malaria transmission is a complex interaction involving several factors, such as the implementation of public health control measures, human migration, drug resistance in parasites, insecticide resistance in vectors, and the involvement of different vector and parasite species [12], the present study adds new information to the relationship between malaria transmission and climatic factors. Our first analysis revealed that the temporal trend of the declining annual incidence of malaria was positively correlated with the declining annual average rainfall; other factors such as the increasing annual maximum, minimum, and mean temperature and relative humidity were negatively correlated. In the second analysis, the Pearson correlation coefficient and *p* value showed that the annual incidence of malaria had a strong negative correlation with annual relative humidity, a weak negative correlation with annual temperature, and a weak positive correlation with annual average rainfall. The annual minimum temperature of 18–19 °C, the maximum temperature of 30–31 °C, the mean temperature of 24–25 °C, rainfall of 100–120 cm, and relative humidity of 55–65% favored malaria cases; hence, during the years with these climatic conditions, malaria cases were reported more frequently. The scatter plots also confirmed the positive correlation of rainfall and the negative correlation of maximum, mean, and minimum temperature and humidity with the annual incidence of malaria. Moreover, the scatter plot analysis narrowed the range of climatic conditions that are most suitable for malaria incidences.

As India has fixed its goal of eliminating malaria by 2030 [40], the influence of such climatic factors on the occurrence of malaria needs to be given sufficient attention. Despite the strong influence of rainfall and humidity and the weak influence of temperature, the ever-low incidence of 0.00010 malaria cases per million population indicates the progression of India toward reducing the annual incidence of malaria and its steady march toward elimination. As the declining rainfall is shown to be positively correlated with declining rates of malaria, the projected future increase in rainfall in India, which is estimated to be from 10% to 72% with an average increase of 24.3% [24], may increase the future incidence of malaria by providing ample breeding sources. The increase in the annual average temperature of India may impact different regions differently. As the increasing temperature will eventually reduce the vector breeding sites, it will negatively influence the annual incidence of malaria. There is a likelihood of an increasing incidence of malaria in the northern subtropical regions where the current annual average temperature is comparatively less than in the southern tropical regions of India. In contrast, the annual incidence of malaria in near future may likely decline in coastal and peninsular regions of India as a result of the increasing annual average temperature where the current annual average temperature is comparatively higher than the rest of India [41]. It has also been reported that the increasing temperature may reduce the transmission of malaria in previously endemic areas [39].

Additionally, the incidence of malaria may increase at higher altitudes and elevated terrains, which are comparatively cooler in the present day [42]. Therefore, due to changing climatic conditions, malaria may vanish from once devastated areas in the southern half of India and at the same time may gain access into newer areas that are not affected by malaria. The areas that are expected to have a higher transmission of malaria are currently at the fringes of transmission. Apart from changes in climate which may alter the prevalence and incidence of malaria, the future spread of malaria may also depend on factors such as ecological and social changes, politics, and economics. Other factors such as improvements in diagnosis, treatment, reporting methodologies, prevention, and control strategies that have evolved over this period may all have considerable influences on the incidence of malaria, apart from the climatic factors. Therefore, an integrated approach is required for the better management of malaria transmission in addition to paying attention to climate-based studies and developing prediction models. Although calculating an average temperature, humidity and rainfall for the whole country with different climatic zones may not reflect the real picture, the average may, however, reflect the overall variations in the climatic factors which can give newer perspectives when correlated with temporal trends of any particular disease. There are certain limitations of this study that need to be mentioned. 1. This study has taken only passively reported cases from the health care facilities for analysis. As underreporting is a major problem in India, the actual case burden may be several-fold higher than that of the reported cases. 2. Even though homogenized climatic data were used in this study, the climatic conditions are not homogenous for a country such as India with vast climatic differences. However, the average annual climatic factors may give a fair overall picture of the average climatic conditions that prevailed in any particular year, which can be used to determine their influence on the incidence of malaria. As climatic conditions may vary among regions, an elaborate spatio-temporal study examining the incidence of malaria in those particular regions needs to be carried out to obtain a clearer role of the climatic factors in determining the incidence of malaria.

## 5. Conclusions

The annual incidence of malaria had a weak negative correlation with annual temperature, a strong negative correlation with relative humidity, and a positive correlation with rainfall. The scatter plot and temporal trend analysis shows that the future increase in temperature coupled with reduced rainfall may reduce the transmission of malaria in previously endemic areas. The hilly, elevated areas may experience more incidences of malaria due to increasing temperatures in future. Focal, prospective studies, sampling selected representative regions, may be conducted to obtain more information on the role of climatic factors on the incidence of malaria.

## Figures and Tables

**Table 1:** The relationship between the square root count of malaria cases and climatic factors. The correlation coefficient shows that, except for annual rainfall, which is positively correlated with the annual incidence of malaria in India, all other factors such as relative humidity, and maximum, minimum, and mean temperature are negatively correlated to varying degrees.

Climatic Factors	Correlation Coefficient	*p* Value	R^2^ Value
Rainfall	0.28	0.028	0.6016
Relative Humidity	−0.43	0.005	0.4812
Minimum Temperature	−0.23	0.078	0.8692
Maximum Temperature	−0.06	0.631	0.274
Mean Temperature	−0.16	0.204	0.673

**Table 2:** The association of climatic factors with the square root count of malaria cases in respective years. The grey shaded row denotes how many years the square root count of malaria cases was reported as less than 1000 correlating with the range of climatic factors. Similarly, the yellow shaded row denotes how many years the square root count of malaria cases was reported to be more than 1000 correlating with the range of climatic factors. The red colored climatic ranges are considered more serious, during which more than 1000 square root counts of malaria cases were reported at maximum instances.

Square Root of Malaria Cases	Temperature (°C)						Rainfall (cm)		RH (%)	
	Minimum		Maximum		Mean					
	Range	Years	Range	Years	Range	Years	Range	Years	Range	Years
<1000	<18	3	29.5–30	2	<24	2	60–80	0	45–50	0
	18–19	7	30.0–30.5	7	24–25	10	80–100	7	50–55	2
	19–20	2	30.5–31.0	5	25–26	3	100–120	7	55–60	1
	20–21	3	31.0–31.5	2	26–27	1	120–140	1	60–65	2
	21–22	1	>31.5 °C	--	--	--	>140	1	65–70	1
	Total	16	Total	16	Total	16	Total	16	Total	6
>1000	<18	4	29.5–30	9	<24	3	60–80	3	45–50	0
	**18–19**	**29**	**30.0–30.5**	**16**	**24–25**	**37**	80–100	8	50–55	6
	19–20	9	**30.5–31.0**	**17**	25–26	4	**100–120**	**20**	**55–60**	**14**
	20–21	2	31.0–31.5	2	26–27	1	120–140	14	**60–65**	**15**
	21–22	1	>31.5	1	--	--	>140	0	65–70	0
	Total	45	Total	45	Total	45	Total	45	Total	35
Total years		61		61		61		61		41

## Data Availability

The datasets are available from the corresponding author. Please contact the corresponding author.
